# Development of a protein energy malnutrition screening tool for older Thais in public residential homes

**DOI:** 10.1017/S1368980021004250

**Published:** 2022-03

**Authors:** Thitima Phodhichai, Warapone Satheannoppakao, Mathuros Tipayamongkholgul, Carol Hutchinson, Siriphan Sasat

**Affiliations:** 1 Doctoral Student in Doctor of Public Health (International Program), Faculty of Public Health, Mahidol University, Bangkok, Thailand; 2 Department of Nutrition, Faculty of Public Health, Mahidol University, 420/1 Ratchawithi RD., Ratchathewi District, Bangkok 10400, Thailand; 3 Department of Epidemiology, Faculty of Public Health, Mahidol University, Bangkok, Thailand; 4 Department of Adult and Gerontological Nursing, Faculty of Nursing, HRH Princess Chulabhorn College of Medical Science, Chulabhorn Royal Academy, Bangkok, Thailand

**Keywords:** Protein energy malnutrition, Screening tool, Older Thais, Residential home

## Abstract

**Objective:**

This study aimed to develop and validate protein energy malnutrition (PEM) screening tool for older adults in public residential homes, and to test its practicality.

**Design:**

This cross-sectional study consisted of two phases: tool development/validation and tool practicality evaluation. In Phase 1, the questionnaire was developed based on literature review and tested for content validity. Older residents were interviewed using this questionnaire to identify potential PEM risk factors. A 24-h recall was used to collect dietary data, and body composition and serum albumin were measured. In Phase 2, practicality of new PEM screening tool was evaluated by intended users. Data were analysed by *χ* ^2^ test, Fisher’s exact test, *t*-test, Mann–Whitney *U* test and multiple logistic regression. Akaike Information Criterion (AIC) was used to estimate the best fit model.

**Setting:**

Four public residential homes in central region, Thailand.

**Participants:**

249 older residents residing in public residential homes and eight intended users.

**Results:**

26·9 % had PEM (serum albumin <3·5 g/dl). According to multiple logistic regression and AIC values, PEM predictors were having pressure ulcer, experiencing significant weight loss and taking ≥ 9 types of medicine daily. These predictors were included in PEM screening tool. Regarding the tool performance test, area under the ROC curve was 0·8 (*P* < 0·001) with sensitivity and specificity of 83·9 and 45·5 %, respectively. For its practicality, eight intended users reported that it was useful and easy to use.

**Conclusions:**

New screening tool may be capable of identifying PEM in older residents, and further testing is required before being recommended for use.

The ageing population is increasing worldwide as a result of declining fertility and improved longevity. Data from the United Nations predict that the proportion of people aged 60 years and older will increase from 12·5 % in 2017 to 20 % in 2050, globally^(1)^. Similarly, data from the Foundation of Thai Gerontology Research and Development Institute, Thailand, reported that the ageing population (aged 60 years and older) increased from 8·4 % of the population in 2010 to 17·1 % in 2017^(2)^. Furthermore, the proportion is projected to increase to 19·9 % in 2037. Therefore, there are many challenges for health care professionals who have to address their needs.

Older adults have special dietary requirements; however, not all of them can achieve an optimal intake. Consequently, this age group is at high risk of protein energy malnutrition (PEM). PEM is defined as a wasting condition in which the body has inadequate protein, energy and/or other nutrients as a consequence of insufficient food and nutrient intake over time^(3–5)^. It is caused by many factors and reflects deteriorating physical and mental health including poor sensory function^(6)^, poor appetite^(5,6)^, poor cognitive function, difficulty chewing or swallowing, restricted mobility, chronic illnesses, poverty, social isolation and other factors^(6)^. PEM can be linked to many serious health outcomes including increased risk of falls^(7–9)^, reduced functional capacity^(7,9)^, increased risk of complications^(10)^, poorer cognitive function^(11)^, poorer quality of life^(12)^, delayed discharge and increased risk of mortality^(9)^. In addition, it imposes an increased financial burden on older adults, caregivers and communities.

Early detection using screening tools is useful to identify older adults who are at risk of malnutrition. Then a proper nutrition intervention can be provided early. Even though many tools have been developed, there is no gold standard method for the early detection of malnutrition in older adults. Moreover, differences in anthropometry, nutritional characteristics and factors contributing to nutritional status are present between older people in different countries^(13)^. These factors limit the adoption of validated screening tools^(14)^. Therefore, many studies about screening tool development and validation have been conducted.

The most widely used nutrition screening tool in Thailand is the Mini Nutritional Assessment (MNA). Some studies have examined the reliability and validity of this existing nutrition screening tool among older Thai people; however, the effectiveness of the MNA has not been confirmed^(15,16)^. Most importantly, although the MNA was translated into Thai (Thai MNA), questions were developed based on the characteristics of older French citizens, which do not fit the Thai context in terms of dietary habits, BMI and other anthropometrical measurements. Indeed, Chumlea stated that the translation of MNA might not be applicable to non-Western countries due to differences in culture, dietary habits or health care system^(17)^. This is one major justification for developing a new screening tool specifically to detect malnutrition among older Thai people. A second justification point for a new nutrition screening tool is that previous Thai-developed nutrition screening tools focussed on patients in hospital settings and were not tailored towards screening older people^(18–20)^. These tools are the Vajira Nutritional Screening Tool, Bhumibol Nutrition Triage and Nutrition Alert Form. Besides, two of them require the results of biochemical tests. Prior to the completion of this study, there was no published nutrition screening tool tailored towards older Thai residents in long-term care facilities, which have limited specialist human resources.

Institutionalised older adults are mostly dependent, disabled, highly afflicted with functional impairments^(21,22)^ and have chronic illnesses^(23)^ that may compromise energy and nutrient adequacy. Even though nutritious meals are served to them, older adults may dislike foods that are provided as they tend to be unpalatable due to limited salt or sugar content; this may be related to unintentional weight loss^(23)^. Additionally, meals may be difficult for older residents to chew and swallow, which possibly causes older people to eat less^(24)^. Moreover, isolation from their families and living in a new environment may lead to psychological stress^(25,26)^, which in turn puts them at a higher risk of PEM^(22)^.

This current study focussed on older Thai people living in public residential homes that were operated by government or provincial administrative organisations. Even though older adults are physically independent upon admission to public residential homes, they may become dependent later in their life. In addition, this older group is poor, lonely or cannot stay with their family. These scenarios might put them at risk of PEM. Also, some research evidence suggested that these residents were mostly dependent (60·3 %) and had health problems (86·8 %) such as hypertension, cognitive impairment, renal disease, depression and other risk factors for PEM^(27)^. Therefore, a nutrition screening tool for early detection of PEM risk among older Thais in residential homes must be developed and validated, and tested for practicality. The objective of this study was to develop and validate such a tool, and to test its practicality.

## Methods

### Study design and participants

This cross-sectional study was carried out in public residential homes from 2016 to 2017. Across Thailand, there were twenty-five public residential homes at the time that this study was conducted. Ten public residential homes were located in the central region followed by six homes in the northeastern, five in the southern and four in the northern regions^(27)^. A simple random sampling technique was used to select 50 % of a total of ten public residential homes in the central region of Thailand. Then five public residential homes were enrolled as study settings. Participants from the five public residential homes were sampled by using the probability proportion to size technique. This multicentre study was divided into two phases: Phase 1 tool development/validation and Phase 2 tool practicality testing.

Participants in Phase 1 were aged 60 years and older and residing in public residential homes of provincial administrative organisations and the Ministry of Social Development and Human Security, Thailand. Older residents who were unconscious, receiving enteral or parenteral nutrition and suffering from critical illness were excluded. Sample size (*n* 469) was determined by using a sample size calculation for a single proportion^(28,29)^. We determined the standard, which was estimated under the normal curve at Type I error = 0·05 with a PEM prevalence of 17%^(27)^ and a margin of error equal to 3·4 % at a CI of 95 %. Owing to the small population, finite population correction for proportions was used to adjust the sample size^(29)^, then 333 older residents were recruited.

Participants in Phase 2 were composed of nurses and care assistants (defined as intended users) in the selected public residential homes. Two intended users per public residential home were recruited for the tool practicality testing. The researcher excluded nurses and/or care assistants who were not on duty during data collection.

### Research assistants

Before starting data collection, research assistants with, or studying for, a university degree in nutrition were recruited via an announcement placed on notice boards in the Faculty of Public Health, Mahidol University. Research assistants were given an overview of the research project and their roles. They subsequently received operation manuals and training about anthropometric measurement, the 24-h recall method and interview technique. The first author monitored and supervised the whole process of data collection.

### Phase 1: tool development/validation

**Fig. 1:**
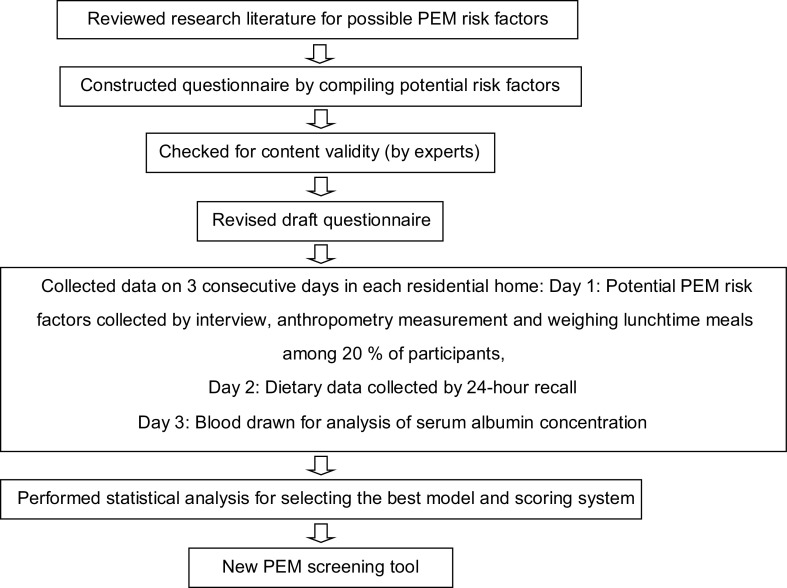
The process of developing and validating the PEM screening tool. Note: PEM, protein energy malnutrition

The process of development/validation of the PEM screening tool is described in Fig. 1. Briefly, it was composed of: (1) A literature review of the possible risk factors associated with PEM in older adults based on previous studies both in Thailand and other countries; (2) Development of the questionnaire by importing the potential risk factors into the questionnaire; (3) Content validity of the questionnaire, which was examined by an expert panel in nutrition and gerontology. After that, the questionnaire was revised based on the experts’ suggestions; (4) data collection was performed; and (5) statistical analysis was conducted for selecting the best model and scoring system. The first author and trained research assistants interviewed participants using a questionnaire that covered potential factors linked to PEM. They were general characteristics (e.g. sex, age, education, income, source of income, current smoking and drinking habit), activities in daily living and health status (e.g. medication, presence of pressure ulcer, oral health, depression, etc.).

Additionally, body composition was determined by using standardised methods and tools by trained research assistants. Measurements included weight, height, calf circumference, mid-upper arm circumference and triceps skin fold thickness. Participants were weighed whilst they were wearing light clothing. Measurements were made to the nearest 0·1 kg by using portable standardised electronic scales (Tanita BC-587). Height was measured to the nearest 0·1 cm by using a stadiometer. Participants’ heels, buttocks, shoulders and head touched the stadiometer, and they looked straight ahead. BMI was then calculated from weight (kg) divided by height in metre squared. Calf circumference was measured to the nearest 0·1 cm using a non-stretchable measuring tape at the widest circumference of the right calf in a sitting position. For mid-upper arm circumference, arm circumference midway between the acromion and olecranon-on the left arm was marked and measured using a non-stretchable measuring tape. Triceps skinfold thickness was measured at the upper arm mid-point mark on the posterior surface of the right arm by Harpenden calliper. Moreover, information regarding weight in kg during the past 6 months (weight recorded in the last 1–6 months) was obtained from each participant’s health record. Then significant weight loss over time (i.e. 5 %, 7·5 % and 10 % weight loss in the previous 1, 3 and 6 months, respectively)^(30)^ was calculated.

Furthermore, dietary intake data were collected using a single 24-h recall by the first author and research assistants with a university degree in nutrition. Information on the type, brand names and amount of food consumed was collected. To increase the accuracy of portion size estimation, household measures and visual aids were used. Due to concerns about participants’ memory (ability to recall), the food weighing method was utilised to validate the outcomes from the 24-h recall method. In this study, a subsample of 20 % of individual lunches was randomly selected for the validation. To validate the 24-h recall by using the food weighing method, we spent 2 d at each residential home. Day 1 was set for weighing lunchtime meals among 20 % of participants. All meal components (served and leftover) were weighed by using a digital kitchen scale (Tanita KD-321) and recorded. On day 2, these participants were interviewed about the food and drink they consumed yesterday using the 24-h recall method. Then intake amounts (for lunchtime meals) from both methods were compared.

To test the new screening tool, a reference standard that is used to diagnose PEM had to be utilised. However, while many criteria are employed to define PEM, there is no universally agreed on reference standard for screening and diagnosing older people with PEM^(31)^. Serum albumin concentration is commonly used for PEM screening, and has some advantages including being easy to measure, relatively cheap and reproducible^(32)^. As a result, serum albumin concentration was used to identify nutritional status; serum albumin < 3·5 g/dl was indicative of a malnourished state^(31)^. For determination of serum albumin concentration, blood was taken by a registered nurse and transferred to serum tubes without anticoagulant. Within 8 h, blood samples were transported at room temperature to a laboratory at the Department of Pathology, Faculty of Medicine, Ramathibodi Hospital, Mahidol University. Serum was separated by centrifugation and stored at −70°C on the same day as sample collection. Then, blood samples were analysed for serum albumin concentration using the dye-binding bromcresol purple technique^(33)^ by laboratory staff.

These factors (i.e. general characteristics, activities in daily living, health status, body composition and energy and macronutrient intake) were analysed as independent variables predicting PEM risk. Statistical analysis was performed for selecting the best model and scoring system to develop a new PEM screening tool.

### Phase 2: tool practicality testing

The PEM screening tool was used by intended users including residential home nurses and care assistants who regularly provided care to the older people. Practicality was assessed to reflect the feasibility of administration and interpretation of this tool. Data were collected by using a self-administered questionnaire consisting of questions about: (i) time taken for each participant to complete the PEM screening tool; (ii) the completeness of items on this tool; (iii) ease of use and (iv) user preferences.

### Data analysis

#### Nutrient analysis

Nutrient intakes were analysed by using INMUCAL-Nutrients Software Version 3.0^(34)^. Then intakes of energy and macronutrients (carbohydrate, protein and fat) were reported.

#### Statistical analysis

After cleaning and coding, the data were analysed by using SPSS version 18.0 (SPSS Inc. Released 2009. PASW Statistics for Windows, Version 18.0 SPSS Inc.). Descriptive statistics were used for explaining participants’ characteristics and general information. The normality of continuous data was examined by the Kolmogorov–Smirnov test. In order to identify potential predictors, the independent variables predicting PEM risk were derived from two main parts because of the large number of independent variables possibly related to PEM. Part 1 was the comparison of differences in characteristics between participants with and without PEM by using the *χ* ^2^ test, Fisher’s exact test, *t*-test or Mann–Whitney *U* test. As for Part 2, factors predicting PEM risk were determined by using simple binary logistic regression. Independent variables with *P* < 0·20^(35)^ and OR ≥ 1·5 were considered to be important factors for PEM. The independent variables which met the aforementioned criteria were entered into multiple logistic regression to identify models for predicting PEM risk. In this step, the Akaike information criterion (AIC) was used for estimating the likelihood of each model to predict PEM. The model that provided the minimum AIC was selected. The performance of the PEM screening tool was explained by sensitivity, specificity, AUC and receiver operating characteristic curve. Sensitivity and specificity were calculated to test the quality of the tool. A receiver operating characteristic curve was determined to discriminate between the residents who were and were not at risk of PEM. For the scoring system, the score of each factor derived from the coefficient of each variable was divided by the lowest *β* value, multiplied by a constant and rounded to the nearest integer to identify participants at risk of PEM. *P* < 0·05 was considered to be statistically significant.

## Results

### Phase 1: tool development/validation

#### Characteristics of participants

**Fig. 2:**
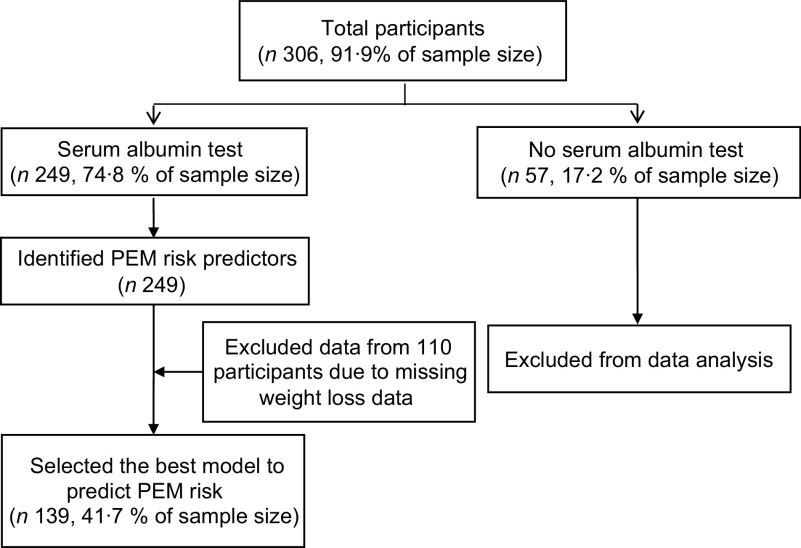
Flow of recruiting participants for tool development/validation phase. Note: PEM, protein energy malnutrition

Initially, 306 older Thai residents from five settings agreed to participate in this multicentre study. However, among the five study settings, fifty-seven participants of one setting declined to have their blood drawn. For that reason, there were serum albumin data (PEM indicator) for only 249 participants. Thus, the response rate was equal to 74·8 % (249 of 306 older residents) as illustrated in Fig. 2, and the data of these 249 participants from four study settings were used. Almost two-thirds of them were female (66·7 %) and had their own income (68·3 %) mainly from individual donors. More than two-fifths were aged 70–79 years (45·4 %). Half of them had completed primary school (55·5 %). Over 80 % were not current smokers, and over 98 % were not current drinkers. Almost 50 % perceived their health status as fair, and 79·5 % did not have depression. The prevalence of PEM (serum albumin < 3·5 g/dl) was 26·9 %.

#### Factors associated with PEM risk

For the univariate analysis (*n* 249), the dependent variable was PEM risk classified by serum albumin level. Independent variables which were possibly associated with PEM (*P* < 0·20) were age, educational level, personal income, perceived health status, received therapeutic diet, difficulty swallowing, activities in daily living score, taking ≥ 9 types of medicine daily, triceps skinfold thickness and experiencing significant weight loss (Table 1). Independent variables which considerably predicted PEM (OR ≥ 1·50) included older age group, educational level, difficulty swallowing, drinking alcohol, having pressure ulcers, taking ≥ 9 types of medicine daily and experiencing significant weight loss (Table 2).

**Table 1:** Factors possibly associated with PEM risk determined by serum albumin

Variables	Total		No PEM		PEM		*P*
	*n*	%	*n*	%	*n*	%	
Sex							0·320*
Male	83	33·3	57	31·5	26	38·2	
Female	166	66·7	124	68·5	42	61·8	
Age (years)							0·140†
Mean	76·99		76·54		78·12		
sd	7·56		7·42		7·89		
Educational level							0·140*
No formal education	43	17·4	30	16·8	13	19·1	
Primary school	137	55·5	98	54·7	39	57·4	
Secondary school	40	16·2	27	15·1	13	19·1	
Diploma/college	5	2·0	3	1·7	2	2·9	
Bachelor and higher	22	8·9	21	11·7	1	1·5	
Personal income							0·050*
No	79	31·7	51	28·2	28	41·2	
Yes	170	68·3	130	71·8	40	58·8	
Smoking							0·820*
Yes	35	14·1	26	14·4	9	13·2	
No	214	85·9	155	85·6	59	86·8	
Drinking							0·300‡
Yes	4	1·6	2	1·1	2	2·9	
No	245	98·4	179	98·9	66	97·1	
Perceived health status							0·130*
Poor	39	15·7	28	15·5	11	16·2	
Fair	122	49·0	94	51·9	28	41·2	
Good	70	28·1	44	24·3	26	38·2	
Excellent	18	7·2	15	8·3	3	4·4	
Presence of pressure ulcer							0·410‡
Yes	17	6·8	11	6·1	6	8·8	
No	232	93·2	170	93·9	62	91·2	
Receive therapeutic diet							0·090‡
No	237	95·2	175	96·7	62	91·2	
Yes	12	4·8	6	3·3	6	8·8	
Number of meals							0·840‡
1	1	0·4	1	0·6	0	0·0	
2	18	7·3	14	7·8	4	5·.9	
3	229	92·3	165	91·7	64	94·1	
Chewing problem							0·630*
No problem	123	49·6	88	48·9	35	51·5	
Had minor problem	108	43·6	81	45·0	27	39·7	
Had major problem	17	6·9	11	6·1	6	8·8	
Swallowing difficulty							0·020*
No	222	89·9	156	87·2	66	97·1	
Yes	25	10·1	23	12·8	2	2·9	
Sense of smell							0·740*
No difference	205	82·3	147	81·2	58	85·3	
Poorer	25	10·0	19	10·5	6	8·8	
Better	19	7·6	15	8·3	4	5·9	
Sense of taste							0·430*
No difference	200	80·3	144	79·6	56	82·4	
Poorer	24	9·6	20	11·0	4	5·9	
Better	25	10·0	17	9·4	8	11·8	
Appetite							0·290*
No difference	157	63·1	114	63·0	43	63·2	
Better	12	4·8	11	6·1	1	1·5	
Poorer	80	32·1	56	30·9	24	35·3	
ADL score							0·010§
Median	20·00		20·00		20·00		
25th, 75th percentiles	19·00, 20·00		19·00, 20·00		19·00, 20·00		
Depression							0·710*
No	198	79·5	145	80·1	53	77·9	
Yes	51	20·5	36	19·9	15	22·1	
Taking ≥ 9 types of medicine daily							0·010*
No	206	84·1	157	87·7	49	74·2	
Yes	39	15·9	22	12·3	17	25·8	
BMI (kg/m^2^)							0·470§
Median	23·21		23·53		22·16		
25th, 75th percentiles	20·47, 26·18		20·52, 26·20		20·23, 26·13		
MUAC (cm)							0·670§
Median	28·30		28·30		28·30		
25th, 75th percentiles	25·50, 30·50		25·60, 30·38		25·00, 31·50		
Triceps skinfold thickness (mm)							0·080§
Median	15·45		16·00		14·00		
25th, 75th percentiles	11·20, 20·00		11·40, 20·90		10·40, 18·20		
Calf circumference (cm)							0·470†
Median	33·00		33·40		32·75		
25th, 75th percentiles	30·80, 35·50		30·75, 35·60		30·88, 35·53		
Experienced significant weight loss (*n* 142)							0·048*
No	73	51·4	61	56·0	12	36·4	
Yes	69	48·6	48	44·0	21	63·6	
Energy intake (kcal)							0·700§
Median	920·40		926·36		916·70		
25th, 75th percentiles	627·47, 1210·14		614·36, 1210·24		628·64, 1210·90		
Carbohydrate intake (g)							0·750§
Median	141·93		140·34		144·14		
25th, 75th percentiles	102·57, 193·58		100·30, 195·06		110·52, 187·29		
Protein intake (g)							0·590§
Median	29·94		30·27		29·44		
25th, 75th percentiles	21·13, 43·05		21·43, 43·84		20·43, 40·68		
Fat intake (g)							0·920§
Median	22·73		22·48		23·13		
25th, 75th percentiles	13·84, 32·56		14·29, 32·22		13·21, 34·38		

Note: ADL, activities in daily living; MUAC, mid-upper arm circumference; PEM, protein energy malnutrition.

* *χ* ^2^ test.

† *t*-test.

‡ Fisher’s exact test.

§ Mann Whitney *U* test.

**Table 2:** Univariate analysis of factors associated with PEM classified by serum albumin using simple binary logistic regression

Variables	Coefficient	*P*	OR	95 %CI
Sex				
Male	0·330	0·267	1·391	0·777, 2·491
Female			1·000	Ref.
Age group (years)				
60–69			1·000	Ref.
70–79	0·263	0·527	1·301	0·576, 2·941
≥ 80	0·434	0·308	1·543	0·671, 4·742
Educational level				
No formal education	2·095	0·052	8·129	0·982, 67·310
Primary school	2·123	0·041	8·357	1·087, 64·279
Secondary school	2·314	0·032	10·111	1·223, 83·598
College	2·639	0·054	14·000	0·952, 205·841
Bachelor and higher			1·000	Ref.
Personal income				
No			1·000	Ref.
Yes	−0·523	0·079	0·593	0·330, 1·063
Smoking				
Yes	−0·071	0·864	0·931	0·412, 2·105
No			1·000	Ref.
Alcohol drinking				
Yes	1·019	0·313	2·769	0·382, 20·064
No			1·000	Ref.
Perceived health status				
Poor	0·675	0·352	1·964	0·474, 8·145
Fair	0·398	0·551	1·489	0·402, 5·517
Good	1·022	0·133	2·778	0·733, 10·529
Excellent			1·000	Ref.
Presence of pressure ulcer				
Yes	0·425	0·422	1·529	0·542, 4·312
No			1·000	Ref.
Receive therapeutic diet				
Yes	−0·701	0·246	0·496	0·152, 1·620
No			1·000	Ref.
Number of meals				
1	−20·234	1·000	0·000	0·000
2	−0·284	0·628	0·753	0·239–2·374
≥ 3			1·000	Ref.
Chewing problem				
No problem			1·000	Ref.
Had minor problem	−0·227	0·451	0·797	0·442, 1·438
Had major problem	0·316	0·563	1·371	0·471, 3·994
Swallowing difficulty				
Yes	1·560	0·038	4·761	1·091, 20·781
No			1·000	Ref.
Sense of smell				
No difference			1·000	Ref.
Poorer	−0·199	0·688	0·820	0·312, 2·157
Better	−0·368	0·529	0·692	0·220, 2·175
Sense of taste				
No difference			1·000	Ref.
Better	−0·640	0·262	0·527	0·172, 1·612
Poorer	0·216	0·637	1·241	0·507, 3·039
Appetite				
No difference			1·000	Ref.
Better	−1·423	0·179	0·241	0·030, 1·923
Poorer	0·067	0·825	1·070	0·588, 1·945
Total ADL score	−0·010	0·922	0·990	0·810, 1·210
Depression				
Yes	0·157	0·651	1·170	0·592, 2·310
No			1·000	Ref.
Taking ≥ 9 types of medicine daily				
Yes	0·802	0·028	2·229	1·091, 4·552
No			1·000	Ref.
BMI				
Normal			1·000	Ref.
Underweight	0·189	0·676	1·209	0·497, 2·941
Overweight	−0·363	0·397	0·696	0·301, 1·611
Obesity	−0·332	0·336	0·718	0·365, 1·410
MUAC	−0·001	0·987	0·999	0·933, 1·071
Triceps skinfold thickness	−0·040	0·096	0·961	0·917, 1·007
Experienced significant weight loss (*n* 184)				
Yes	0·730	0·077	2·075	0·924, 4·657
No			1·000	Ref.
Energy intake	0·000	0·541	1·000	0·999, 1·000
Fat intake	0·001	0·897	1·001	0·982, 1·021
Carbohydrate intake	−0·001	0·476	0·999	0·994, 1·003
Protein intake	−0·007	0·386	0·993	0·977, 1·009

Note: Ref., reference category; ADL, activities in daily living; MUAC, mid-upper arm circumference; PEM, protein energy malnutrition.

Experienced significant weight loss (i.e. 5 %, 7·5 % and 10 % weight loss in the previous 1, 3 and 6 months, respectively, was calculated^(30)^.

These variables were then incorporated in to multiple logistic regression, in order to select the best model to predict PEM risk. However, data from 110 participants were excluded due to missing weight loss data. Consequently, data from 139 participants were utilised to develop the PEM screening tool. In this step, the AIC was used for estimating the likelihood of a model to predict PEM. The model providing minimum AIC, which contained significant factors from the literature review, was selected. The model that included taking ≥ 9 types of medicine daily, having pressure ulcers and experiencing significant weight loss was used for developing the PEM screening tool (Table 3).

**Table 3:** Models predicting the occurrence of PEM classified by serum albumin

Variables	Model 1	Model 2	Model 3	Model 4	Model 5
Educational level	✓				
Swallowing difficulty	✓				
Taking ≥ 9 types of medicine daily	✓	✓	✓	✓	✓
Limited in ADL			✓		
Presence of pressure ulcer		✓			
Experienced significant weight loss		✓	✓		
Low triceps skinfold thickness				✓	
Loss of appetite				✓	
*n*	241	141	139	139	139
AIC	267·2	122·1	120·1	118·1	120·8

Note: ADL, activities in daily living; PEM, protein energy malnutrition.

#### Scoring system

The possibility of scoring ranged from 0 to 7 points. The score of each factor derived from the coefficient of each variable was divided by 1·11 (lowest *β* value), multiplied by the constant 2 and rounded to the nearest integer. The scoring system of the PEM screening tool is shown in Table 4.

**Table 4:** Scoring system of the PEM screening tool

Risk factor	Coefficient	OR	95 % CI	*P*	Point
Presence of pressure ulcer	1·72	5·6	1·2, 26·7	0·03	3
Significant weight loss	1·11	3·0	1·1, 8·3	0·03	2
Taking ≥ 9 types of medicine daily	1·32	3·8	1·2, 12·2	0·03	2

Note:PEM, protein energy malnutrition.

The PEM screening tool consisted of three questions including whether or not a participant had a pressure ulcer, experienced significant weight loss or took ≥ 9 types of medicine. The risk level was defined as at risk of PEM and not at risk of PEM. The sensitivity and specificity of the predicted model are shown in Table 5. It indicated that a participant was at risk of PEM if they answered ‘Yes’ to only 1 out of 3 questions, which equated to a score of 2.

**Table 5:** Score, sensitivity and specificity of predicted model

Score	Sensitivity	95 % CI	Specificity	95 % CI
−1·0	1·000		0·000	
2·0	0·839	0·751, 0·927	0·455	0·383, 0·527
5·0	0·290	0·238, 0·342	0·873	0·838, 0·908
7·0	0·258	0·209, 0·307	0·909	0·879, 0·939
9·0	0·097	0·067, 0·127	0·982	0·969, 0·995
12·0	0·000	0·000, 0·000	0·991	0·982, 1·000

**Fig. 3:**
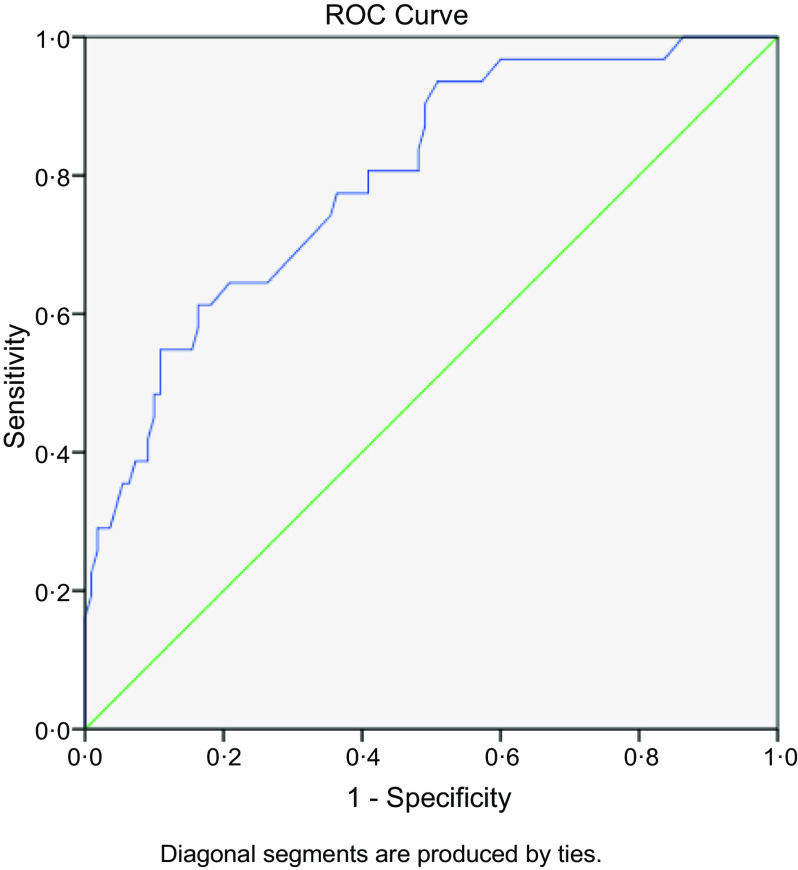
Receiver operating characteristic (ROC) curve of PEM screening tool. Note: PEM, protein energy malnutrition.

The AUC, sensitivity and specificity of the PEM screening tool are described in the receiver operating characteristic curve (Fig. 3). The receiver operating characteristic curve was used to determine the cut-off point of the screening tool and determine the scoring system. The AUC was 0·795 (*P* < 0·001), meaning there is 79·5 % chance that this model is able to distinguish between PEM and no PEM groups. The best cut-off point was 1·0. It provided the best sensitivity and specificity (83·9 (95 % CI 75·1, 92·7) and 45·5 (95 % CI 38·3, 52·7), respectively).

### Phase 2: tool practicality test

Participants of this phase were eight intended users from four public residential homes, namely four nurses and four care assistants who had more than 1-year work experience. These intended users were asked to interview thirty-nine residents (eight males and thirty-one females) of their residential homes by using the PEM screening tool. Collection of data using this PEM screening tool could be completed within 5 min (data not shown). Furthermore, the eight participants from the four different settings agreed that the screening tool was useful (100·0 %), easy to use (87·5 %) and easy to interpret (100·0 %). They also reported that the questions were easy for older residents to understand. Yet, the clarity of one question (regarding significant weight loss) needed to be improved.

## Discussion

Institutionalised older adults are at risk of PEM. Early screening for PEM risk factors is important. This study aimed to develop a PEM screening tool for older adults in public residential homes and to validate and test the practicality of this tool. Three hundred and six older residents were recruited. Fifty-seven from one setting were excluded from data analysis due to missing serum albumin data, leaving 249 older participants from only four settings. The study response rate was 74·8 %. As reviewed, a response rate of approximately 60 % is considered to be acceptable^(36)^. Thus, the response rate in this study was more than satisfactory. Furthermore, there were no statistically significant differences between the participant group (*n* 249) and the excluded group (*n* 57) in terms of seven out of eight of their general characteristics, namely sex (*P* = 0·080), age (*P* = 0·290), educational level (*P* = 0·220), income (*P* = 0·060), smoking behaviour (*P* = 0·150), drinking habit (*P* = 1·000) and perceived health status (*P* = 0·110).

Regarding a biomarker that makes use of the definition of PEM in this study, serum albumin concentration was selected to test against the new screening tool because serum albumin concentration is easy to measure, relatively cheap, reproducible and commonly used for PEM screening^(32)^. Even though it has a long half-life and its level may be affected by infection, burns, fluid overload, hepatic failure and nephrotic syndrome^(37)^, serum albumin is a mainstay in the screening and monitoring of malnutrition^(38)^. Furthermore, to reduce these confounders, older residents who had all of the aforementioned health problems, except infection, were excluded from this study; thereby increasing the likelihood that serum albumin concentration provided a truer reflection of nutritional status.

Using a serum albumin cut-off of < 3·5 g/dl^(31)^, around one quarter (26·9 %) of participants had PEM. Due to a lack of research using serum albumin to assess nutritional status in older Thais living in public residential homes, it is difficult to draw comparisons. An extensive review of the literature revealed only one study that measured serum albumin concentration. The study was conducted in 1997 by Charoonruk and it examined the nutritional status of 139 older Thai residents in one public residential home^(39)^. The prevalence of PEM in Charoonruk’s study 23 years ago was 14 %. Nevertheless, when the same setting as that used by Charoonruk was included in this study, the PEM prevalence was slightly lower than that reported by Charoonruk (12·7 *v*. 14·0 %, respectively). Thus, PEM prevalence in Thailand may not have markedly increased over two decades. We recruited participants from four residential homes, whereas Charoonruk collected data from only one setting. The current study uncovered wide variation in PEM prevalence (12·7 % to 37·2 %) across the four settings (data not shown). However, it is recognised that PEM has been a common problem among older Thai residents in public residential homes for some time, and prevention and treatment of PEM continue to challenge health professionals.

In the development/validation phase, factors associated with PEM risk were firstly investigated by univariate analysis. All independent variables that were probably linked with PEM risk (*P* < 0·20 or OR ≥ 1·50) were selected as candidate PEM predictors for multiple logistic regression. As only 139 out of 249 older residents had complete data, it should be noted that the reduced number of participants might have affected the predictors of PEM risk.

The AIC, which was applied in this study, is generally considered to be the first model selection criterion to use in practice^(40)^. Even though the model with the lowest AIC is considered to be the best model (contained taking ≥ 9 types of medicine daily, low triceps circumference and loss of appetite), we selected the second lowest AIC model as the significant predictors for this PEM screening tool. The reason being that the main goal was to produce a screening tool which is easy to use, concise, economical and usable by people without nutrition expertise^(41)^. Measurement of triceps skinfold circumference (in the model with the lowest AIC) requires a specific and high-cost instrument, which may be difficult for residential home staff to access. Well-trained and experienced staff are also required to take accurate skinfold measurements. Furthermore, a screening tool with multiple items must meet standards of reliability^(42)^.

The AUC was 0·795, which provided the best sensitivity (83·9 %) and specificity (45·5 %). The sensitivity and specificity of a screening test are characteristics of the test’s performance at a given cut-off point (criterion of positivity)^(43)^. Ideally, a test should provide high sensitivity and specificity^(43)^. PEM is a health problem which can be prevented, so we focussed on sensitivity because this test is more likely to correctly identify older adults who are at risk, confirm risk and then provide a nutrition intervention. Furthermore, high sensitivity is important where an undetected condition has serious consequences but is treatable^(43–45)^. The AUC is a measure of the cut-off accuracy of a test and the figure obtained in this study (0·795) indicated that the tool performed well in distinguishing older adults with PEM and without PEM^(46)^. PEM screening tools can be easy to administer, but accuracy remains essential.

Nutrition screening tools contain a variety of risk factors, in terms of type and number^(47)^. Most screening tools are based on basic questions covering weight loss, current BMI, dietary intake, disease severity or some other measurement^(44,48,49)^. Some nutrition screening tools include physical examination, such as Subjective Global Assessment Test, which is dependent on the availability of a health professional who is a skilled and experienced observer^(47)^. In reality, many nutrition screening tools often require experienced clinicians and dietitians or longer periods of time^(32)^ to collect data and/or interpret outcomes. As is the case in low- and middle-income countries in general, residential homes in Thailand do not employ full-time nutritionists or dietitians to provide food services and nutrition care. Hence, early detection of PEM is rare.

In this study, having pressure ulcers (OR 5·6 (95 % CI 1·2, 26·7)), experiencing significant weight loss (OR 3·0 (95 % CI 1·1, 8·3)) and taking ≥ 9 types of medicine daily (OR 3·8 (95 % CI 1·2, 12·2)) were associated with PEM occurrence, and these factors were included in the PEM screening tool. This outcome is in line with other studies conducted in clinical, community or long-term care settings in other countries, which also demonstrated that older adults with pressure ulcers^(50)^, polypharmacy^(51,52)^ and weight loss^(53)^ had a higher risk of PEM and/or malnutrition. A cross-sectional multicentre study by Bonetti and colleagues examined factors related to malnutrition among patients admitted to twelve hospitals in northern Italy. Presence of pressure ulcer was significantly associated with malnutrition (OR 4·95 (95 % CI 2·63, 9·31), according to multivariate logistic regression)^(50)^. Additionally, greater use of medicine can lead to malnutrition. Nevertheless, there is no consensus on the definition of greater use of medicine or polypharmacy. According to previous publications, the number of medicines required to be considered polypharmacy varies from more than 4 to 10^(13,54,55)^. Therefore, the number of medicines that we used as the cut-off point varied from 4 to 10. Based on the outcomes of binary logistic regression and multiple logistic regression, taking more than or equal to nine types of medicine was a predictor of PEM risk in this study. Medeiros *et al.* performed a cross-sectional study among older adults living in seventeen nursing homes in Brazil to examine factors linked to frailty and malnutrition. They found that older adults taking more medicines had a higher chance of frailty and malnutrition (adjusted PR 1·016 (95 % CI 1·006, 1·027)). Medeiros *et al.* proposed that the relationship between greater medicine use and malnutrition may be due to medication side effects, including appetite and sensory alterations^(52)^. As for weight loss, it seems to be allied with malnutrition in several age groups. de Aquino and Philippi investigated malnutrition risk factors among Brazilian hospital patients who were aged 18–64 years old. The strongest predictor was weight loss (OR 58·03 (95 % CI 18·46, 182·41))^(53)^.

However, compared to other nutrition screening tools^(18–20)^ developed in the Thai context, the factors that predicted PEM in this screening tool were different. The Vajira Nutritional Screening Tool, which was designed to assess the nutritional status of hospital patients, is composed of four significant factors including BMI < 18·5 kg/m^2^, weight loss within 3 months, decreased food intake within a week and chronic illnesses or surgery^(18)^. Regarding the Nutrition Alert Form developed by Komindr *et al.* ^(19)^, PEM predictors are arm span, BMI, albumin or total lymphocyte count, weight change within 4 weeks, body shape, gastrointestinal problems, food accessibility and morbidities. Differences in the characteristics of participants in each setting (hospital-based or residential home-based) might explain the variation in PEM risk factors.

Questions in this PEM screening tool also differ from the Mini Nutritional Assessment Short-Form (MNA-SF) which includes questions about appetite loss, weight loss over 3 months, mobility, acute disease, BMI and neuropsychological problems^(56)^. MNA-SF has some advantages for use in long-term care facilities, for example, it does not require a laboratory test. However, to our knowledge, some factors in the MNA-SF might be of limited use for screening residents in a long-term care facility. For example, its questions about neuropsychological problems or psychological stress should not rely on only residents’ self-evaluation of themselves, but also require judgment from specialists in this field. Apart from the differences between this new screening tool and others mentioned above, as noted, one factor predicting PEM risk that has been commonly found in all screening tools is a loss or change in weight in these vulnerable groups. Consequently, weight loss or weight change should be a concern.

Residential homes provide services at all levels of care because the majority of residents have chronic health problems and need moderate to high levels of care. Thus, reliable and valid assessment of instruments and adequate health care services are required in order to appropriately assess and address these needs^(57)^. Nutrition screening or assessment in secondary and tertiary care is widely considered to be a useful tool to identify older people who are at risk of malnourishment^(47)^. Some evidence suggests that screening prior to admission to care homes may also be beneficial^(48,58)^. Green and Watson hinted that earlier identification might help to reduce the malnutrition trajectory and the negative outcomes associated with poor nutritional status^(47)^. The screening process should be simple, acceptable to intended users and older participants, and should not require any nutrition expertise.

The main strength of this study was its inclusion of several residential homes (four out of ten) located in the central region of Thailand. This helped to expand the number of participants that met the eligibility criteria. The participants were also representative of the older adults living in public residential homes in this region. Furthermore, this screening tool had high sensitivity and AUC. Consequently, it was able to screen older residents in public residential homes who were at risk of PEM. Additionally, this screening tool was accepted by intended users, namely nurses and care assistants, as it is easy to use and interpret.

Some limitations are presented. The first is related to incomplete secondary data used for predicting the potential PEM risk factors. For example, weight loss data were not recorded regularly in all study settings. Therefore, the analysis of PEM predictors by multiple logistic regression included fewer than expected participants with complete data. Hence, due to incomplete data, further testing of this proposed PEM risk screening tool is required. Secondly, in this multicentre study, albumin was used as a biomarker of PEM. It is generally acknowledged that albumin is not the most sensitive biomarker of malnutrition due to a long half-life and potential interference from several factors^(59)^. However, older residents who had potentially confounding health problems, with the exception of infection, were excluded from participating. Furthermore, research has demonstrated that serum albumin remains a suitable indicator for screening and monitoring malnutrition^(38)^. Nevertheless, using an imperfect reference standard may affect estimates of diagnostic accuracy. In light of this, the ranges of bounded values for estimated sensitivity and specificity are presented to explain their uncertainty^(60)^. Another limitation concerns the collection of dietary intake data covering only 1 d. A single day dietary account might not be representative of an individual’s habitual dietary consumption. Nonetheless, it is suitable for estimating the average intakes of a group or population^(61)^. Using the 24-h recall method to collect information on dietary intake from older adults can be questionable, due to respondent memory lapses. In this study, this method was used because it presents a low burden to participants and does not affect their dietary habits. Household measures and visual aids were used to help assist participants to more accurately recall portion sizes. Moreover, we weighed 20 % of participants’ meals at lunchtimes. It was found that average intake amounts ascertained by weighing and dietary recall were comparable (*r* = 0·61, *P* < 0·001), thereby indicating that the 24-h recall data were acceptable in terms of its accuracy in estimating the amounts consumed. As noted, in this study, the median energy intake was lower than the 2003 and 2020 Thai recommendations for these age groups^(62,63)^. It was also somewhat lower than the energy intakes reported in the Thai National Health Examination Survey IV^(64)^. The relatively low energy intakes in this study may reflect the study population group, which only included older adults residing in residential care homes, who tend to have health conditions which can adversely affect their dietary intakes^(21–23)^.

In conclusion, intended users reported that the PEM screening tool developed in this study was useful and easy to use and interpret. Besides, the questions in this screening tool were easy for older residents to understand. This screening tool could be useful for detecting PEM among older adults who live in public residential homes; however, further testing of the tool is required before it can be recommended for use.
